# Crystallization Kinetics of Phosphonium Ionic Liquids: Effect of Cation Alkyl Chain Length and Thermal History

**DOI:** 10.1021/acs.jpcb.4c01720

**Published:** 2024-06-26

**Authors:** B. Yao, V. Morales Alvarez, M. Paluch, G. Fedor, S. McLaughlin, A. McGrogan, M. Swadźba-Kwaśny, Z. Wojnarowska

**Affiliations:** † Institute of Physics, The University of Silesia in Katowice, 75 Pułku Piechoty 1A, Chorzów 41-500, Poland; ‡ The QUILL Research Centre, School of Chemistry and Chemical Engineering, The Queen’s University of Belfast, David Keir Building, Stranmillis Rd, Belfast, NI BT9 5AG, U.K.

## Abstract

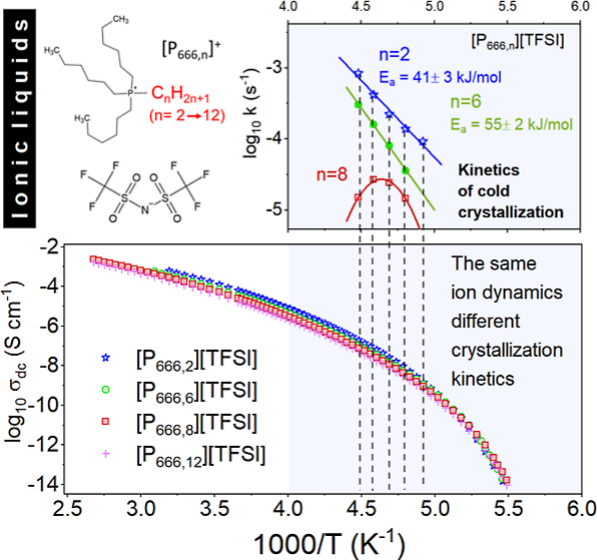

The effects of alkyl chain length on the crystallization kinetics and ion mobility of tetraalkylphosphonium, [P_666,*n*_][TFSI], (*n* = 2, 6, 8, and 12) ionic liquids were studied by differential scanning calorimetry (DSC) and broadband dielectric spectroscopy (BDS) over a wide temperature range. The liquid–glass transition temperature (*T*_g_) and ion dynamics examined over a broad *T* range were almost insensitive to structural modifications of the phosphonium cation. In contrast, the crystallization kinetics were strongly affected by the length of the fourth alkyl chain. Furthermore, the thermal history of the sample (cold vs melt crystallization) significantly impacted the crystallization rate. It has been found that the nature of crystallization phenomena is the same across the homologous series, while the kinetic aspect differs. Finally, electric conductivity in supercooled liquid and crystalline solid phases was measured for all samples, revealing significant ionic conductivity, largely independent of the cation structure.

## Introduction

Ionic liquids (ILs), salts that melt below 100 °C, have been of interest to chemists for decades, although their applications have changed over time. Recently, ILs have been gaining interest as functional materials equipped with fascinating physicochemical properties that arise from strong Coulombic interactions between ions in the liquid phase. Ionic conductivity enables efficient charge transport and facilitates various electrochemical applications.^1^ The notable chemical and thermal stability, together with negligible vapor pressure and a wide liquidus range, make them attractive candidates across applications in physics and material science.^2^ In order to expand the liquidus range of ILs, their ions have been designed to disrupt crystallization (viz., low symmetry and low and dispersed charges). This resulted in the unique phase behavior of ILs, fueling fundamental studies and inspiring new applications.

In applications of ILs as electrolytes in energy storage devices, it is crucial to maintain their liquid state under various thermodynamic conditions, including elevated pressure. Therefore, ILs that do not crystallize over a wide temperature and pressure range are valuable in this context.^3,4^ In contrast, ILs that crystallize reproducibly on cooling or reheating are desired for purification strategies and as phase change materials in energy storage and thermal management applications.^5,6^ Furthermore, a subclass of partially ordered ILs, called organic ionic plastic crystals (OIPCs), serve as relatively conductive solid-state electrolytes, which provide better contact with electrodes during volume change than brittle solid electrolytes while preventing the leakage problems associated with liquid electrolytes.^7,8^

The wealth of applications reliant on phase control motivates fundamental studies into liquid–glass transitions^9^ and the crystallization of ILs. However, the understanding of crystallization mechanisms, kinetics, and factors controlling the crystallization tendency of ILs is still incomplete.^10−13^ The studies of imidazolium, pyrrolidinium, and ammonium ILs have shown that altering alkyl chain length can change their dynamic properties and tendency toward crystallization.^14−16^ In particular, the size of the cation side group influences the nature of intermolecular interactions in ILs^17−19^ and affects the diffusion mechanism and ion transport.^20−22^

Studies on the crystallization kinetics and charge transport mechanisms of quaternary phosphonium ILs have not been reported to date.^23^ This is surprising, given the excellent material properties of phosphonium ILs: this class of ILs is especially interesting due to being less viscous and more conductive when compared with their ammonium analogues,^24,25^ showing a tendency to form OIPCs,^26^ and displaying rich nanostructuring arising due to the amphipathic separation of polar and nonpolar domains on the cation.^23,27−31^

In this work, we investigate the crystallization tendency and ion dynamics of phosphonium ILs with a trihexyl(alkyl)phosphonium cation, [P_666,*n*_][TFSI], altering the length of a single side chain (*n* = 2, 6, 8, and 12). To examine the crystallization ability of these materials, a series of isothermal time-dependent measurements have been performed using differential scanning calorimetry (DSC) and broadband dielectric spectroscopy (BDS). This approach enables us to determine the crystallization rate at various temperatures and establish the link between the length of the alkyl chain in the cation and the ordering tendency at different thermodynamic conditions. The effect of the sample’s thermal history on the crystallization rate has also been investigated.

## Experimental Method

### Materials

**Figure 1:**
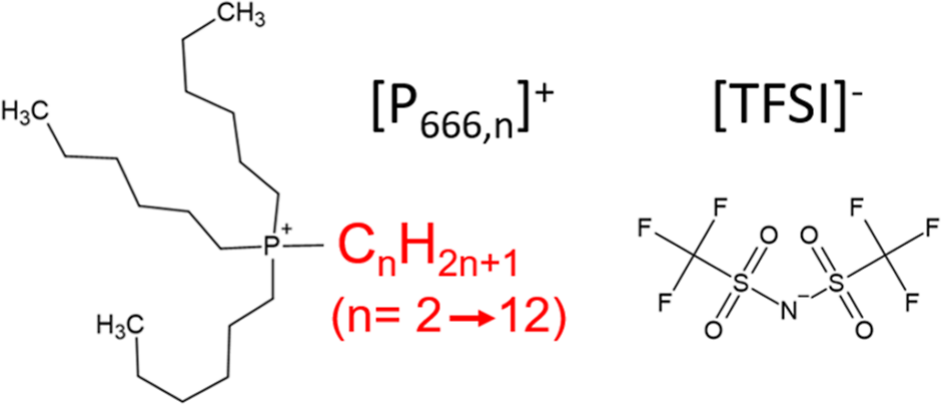
Chemical structures of studied ILs containing the cation [P_666,*n*_]^+^ (*n* = 2, 6, 8, and 12) and [TFSI]^−^ anion.

The synthetic procedure for trihexyl(alkyl)phosphonium bis(trifluoromethylsulfonyl)imide, [P_666,*n*_][TFSI] (*n* = 2, 6, 8, and 12), is reported in our earlier paper.^32^ A generalized structure of the homologue series studied here is shown in Figure 1, and the physiochemical properties of each homologue are collected in Table 1. The water content was determined using a coulometric Karl Fischer titration (899 Coulometer, Metrohm).

**Table 1:** Molecular Weight (*M*), Water Content (*wt*), Purity, and Characteristic Temperatures (Glass Transition Temperature *T*_*g*_, Crystallization Temperature *T*_*c*_, and Melting Point *T*_*m*_) of Studied ILs

ILs acronym	*M* (g/mol)	*wt* (ppm)	purity (%)	*T*_*g*_ (K)		*T*_*c*_ (K)	*T*_*m*_ (K)
				DSC	BDS		
[P_666,2_][TFSI]	564.71	200	>98	185.6^a^	182.3	224.1^b^	246.1^b^
				182.8^b^			
[P_666,6_][TFSI]	620.81	600	>98	184.2^a^	181.4	233.7^b^	258.2^b^
				183.3^b^			
[P_666,8_][TFSI]	648.87	120	>98	182.9^a^	180.5		
				180.3^b^			
[P_666,12_][TFSI]	704.97	680	>98	182.3^a^	181.2		
				180.4^b^			

a 10 K/min.

b 1 K/min.

### DSC

Calorimetric measurements were performed using a Mettler-Toledo DSC1STAR instrument with a liquid nitrogen cooling accessory and an HSS8 ceramic sensor with 120 thermocouples. The nitrogen flow was maintained at 60 mL min^–1^. Indium and zinc standards were used to calibrate the temperature and enthalpy. The samples were measured in aluminum crucibles having a capacity of 40 μL shut with a punctured cover. To ensure repeatability and precision, a fresh sample was prepared for each experiment and cycled at least three times.

### BDS

The conductivity measurements at ambient pressure were performed using a Novo-Control GMBH Alpha dielectric spectrometer over a wide temperature and frequency range (10^–1^ to 10^7^ Hz). The Quattro system controlled the temperature with an accuracy of 0.1 K. During the measurement, the sample was held between two stainless steel electrodes with a 15 mm diameter and a 0.2 mm quartz ring to maintain a stable separation between the electrodes.

The isothermal conductivity studies were carried out at 223, 218, 213, 208, and 203 K in the supercooled liquid state. The ILs were first cooled to a glassy state for cold crystallization studies. Immediately after amorphization, the temperature was increased to the given *T*_c_, and the real part of complex electric conductivity σ′(*f*) was recorded under isothermal conditions, at specified time intervals throughout the crystallization process. For melt crystallization, the liquid sample was supercooled to the crystallization temperature, and then σ′(*f*) spectra were recorded at the same predetermined time intervals during the crystallization process.

## Results and Discussion

### Glass-Forming Study by Differential Scanning Calorimetry

**Figure 2:**
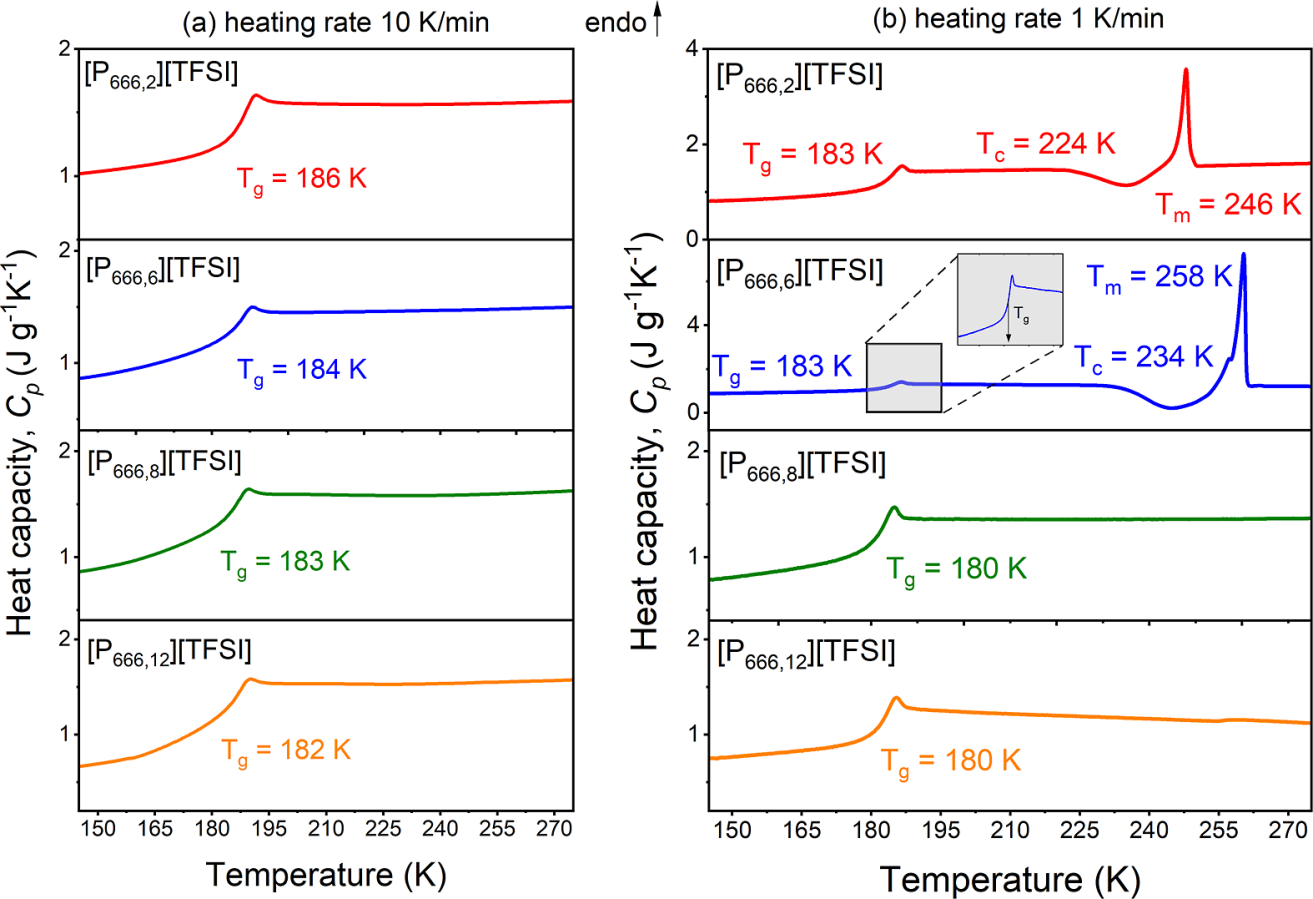
DSC traces of [P_666,*n*_][TFSI] ILs (*n* = 2, 6, 8, and 12) at different heating rates of 10 K/min (a) and 1 K/min (b).

Thermal analysis allows for an understanding of the material properties as a function of temperature. In this work, DSC was used to characterize the thermal properties of a homologous series of [P_666,*n*_][TFSI] ILs, investigating their glass-forming ability and crystallization tendency. Two different temperature ramping rates were used: a standard rate of 10 K/min and a slower rate of 1 K/min. It has been discovered that all the studied ILs could be vitrified on cooling, even at the slow cooling rate of 1 K/min (see Supporting Information, Figure S1). Consequently, in all cases, the subsequent heating curve featured a step-like increase in heat capacity identified with the glass transition (Figure 2). Glass transition temperatures (*T*_*g*_) were found to decrease slightly with elongating cationic alkyl chains and decrease with decreasing ramp rates, but all remained within a narrow range of *T*_*g*_ = 180–186 K (Table 1). In other words, decreasing the alkyl chain length in [P_666,*n*_][TFSI] systems has not resulted in observable changes in the thermal characteristics. This contradicts the behavior of imidazolium-based ILs, which showed a decrease in *T*_g_ for materials with a shorter alkyl substituent.^17,20^ None of the ILs showed a crystallization tendency when a 10 K/min heating rate was applied. At a scan rate of 1 K/min, *T*_*g*_ values were lower, which agrees with the kinetic nature of the vitrification process, and cold crystallization exotherms were recorded for [P_666,2_][TFSI] (*T*_*m*_ = 224.1 K) and [P_666,6_][TFSI] (*T*_*m*_ = 233.7 K). Cold crystallization of the *n* = 2 homologue took place at a temperature 10 K lower than for *n* = 6 homologue, and no cold crystallization was observed for the *n* = 8 or 12, which indicated an increased tendency toward cold crystallization in ILs with short alkyl chain lengths.

Controlling the degree of crystallinity and crystallization rate on cooling and heating is crucial for applications of ILs, which inspired subsequent kinetic studies.

### Kinetics of Isothermal Crystallization of [P_666,2_][TFSI] by DSC

**Figure 3:**
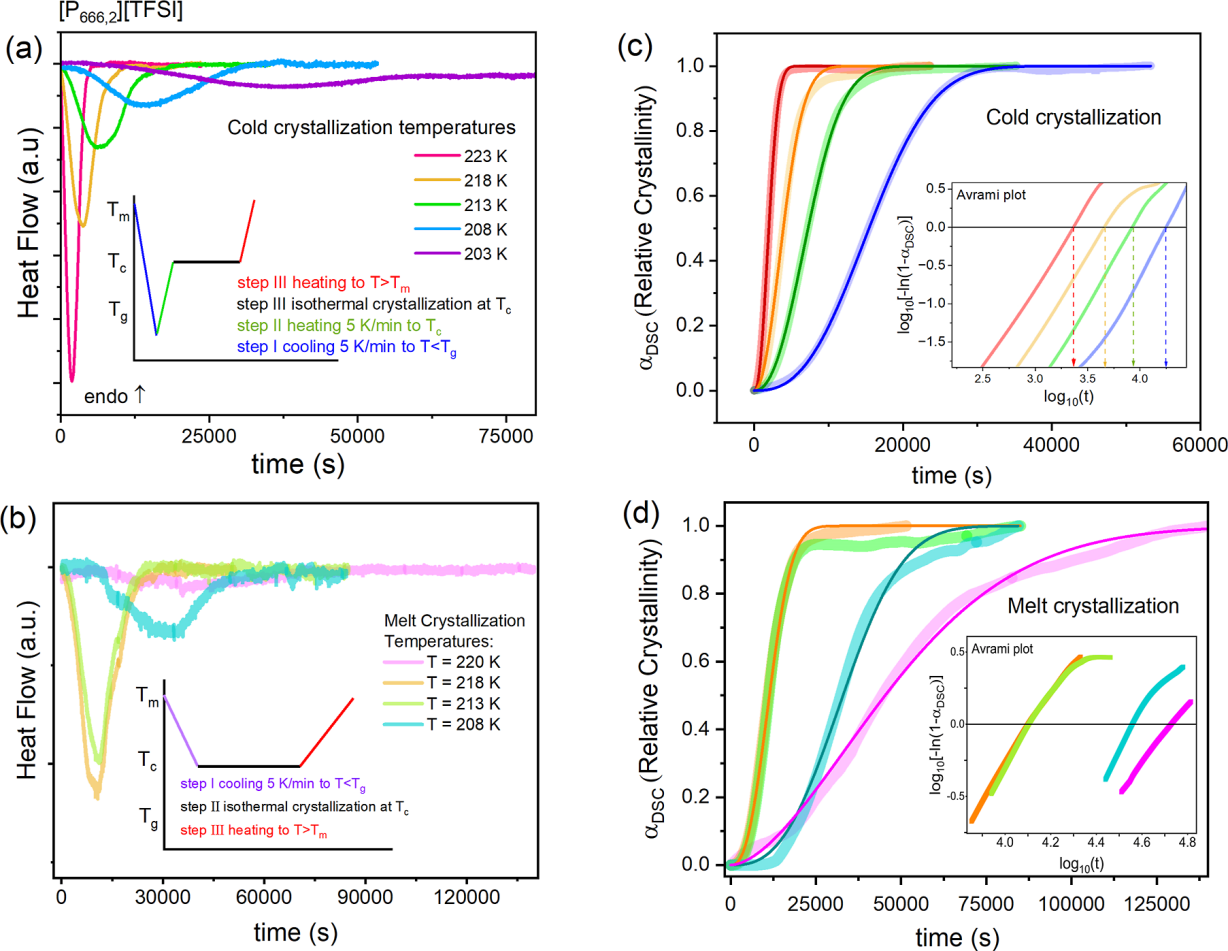
Isothermal crystallization data of [P_666,2_][TFSI] measured by DSC. Heat flow as a function of time during isothermal cold crystallization (a) and melt crystallization (b). Relative crystallinity α_DSC_ versus crystallization time for cold crystallization (c) and melt crystallization (d). Solid lines in panels (c,d) are Avrami fits in terms of eq 2. Insets are Avrami plots of the dependence log[−ln(1 – α_DSC_(*t*))] versus log(*t*) for each isothermal crystallization.

The kinetics of crystallization of [P_666,2_][TFSI] were studied under both isothermal cold crystallization and isothermal melt crystallization conditions. For this purpose, time-dependent DSC measurements were performed at five different temperatures: 223, 218, 213, 208, and 203 K. To monitor the cold crystallization, the sample was first cooled at 5 K/min from RT (303 K) to the temperature below *T*_*g*_ (168 K) and then heated at the same rate to the desired temperature (*T*_*c*_). The sample was kept at *T*_*c*_ until the crystallization process was finished and subsequently heated to the melting point (see the inset of Figure 3a). The isothermal melt crystallization was studied by cooling the sample from room temperature (RT) to *T*_c_ (see the inset of Figure 3b) and holding it at this temperature until the crystallization process was finished, followed by heating above the melting point (see the inset of Figure 3b). The heating scans performed after each isothermal step are presented in Figure S2. Since the *T*_m_ and the enthalpy of the melting process (Δ*H*_m_ = 25.4 J/g) are the same for all scans, it can be assumed that a single polymorphic form is obtained in both cold and melt crystallization. Furthermore, the lack of liquid–glass transitions indicates complete crystallization. The only exceptions to these rules are the cold crystallization at 203 K, where Δ*H*_m_ = 14.6 J/g suggests that 57% of the sample has crystallized, and the melt crystallization at 220 K, where Δ*H*_m_ = 23.75 J/g suggests that 93.5% of the sample has crystallized (Table S1).

The exothermic peaks of isothermal crystallization measured in two different procedures are presented in Figure 3a,b. The cold crystallization maxima shift toward short time scales as *T*_c_ increases, demonstrating that temperature is a major factor controlling cold crystallization. In contrast, for [P_666,2_][TFSI] crystallization monitored right after cooling from RT, there was a maximum in crystallization time scale recorded as a function of temperature: the shortest crystallization was recorded at 218 and 213 K (nearly identical exotherms, see Figure 3b), then at 208, and finally at 220 K. The melt crystallization at 223 K was too slow to be monitored in an available experimental time frame.

By integrating exothermic peaks obtained during the crystallization process, the relative degrees of isothermal crystallization, α_DSC_, at different temperatures could be determined by eq 1
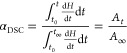
1where *t*_0_ and *t*_∞_ are the times at which crystallization begins and ends, respectively, d*H*/d*t* represents the rate of heat evolution, and *A*_t_ and *A*_∞_ denote the areas under the normalized DSC curves for partially and fully crystalline samples.

The time evolutions of isothermal cold and melt crystallization α_DSC_ are presented in Figure 3c,d, respectively. All α_DSC_(*t*) curves, irrespective of the type and temperature of crystallization, had a sigmoidal shape and reached unity when crystallization was completed. To gain a generalized understanding of the crystallization kinetics of [P_666,2_][TFSI], it has been attempted to fit the experimental data with two theoretical models. First, the Avrami equation (eq 2)^33,34^ has been applied^35^
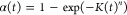
2where *K* = *k*^*n*^ is a crystallization rate constant, which relies on the crystallization temperature and geometry of the material, *n* denotes the Avrami exponent that is connected to the time dependence of the nucleation rate and crystallization dimensionality, and ideally *n* would be between 1 and 4.^36,37^ Typically, when *n* values approach 2, it suggests a two-dimensional growth of crystals, whereas *n* values closer to 3 or higher, as observed in the case of composites, are indicative of heterogeneous nucleation followed by three-dimensional growth.^38,39^ The fitting of the Avrami function to the DSC experimental results is displayed as lines in Figure 3c,d. The Avrami model does not describe the experimental data satisfactorily over the entire time range, which is not surprising and has been many times documented in the literature.^40^ To determine the values of the Avrami parameters, the log form of eq 2 was used for preparing the so-called Avrami plot (eq 3)
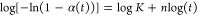
3

According to this equation, log[-ln(1-α_DSC_(*t*))] should depend linearly on log(*t*). Using a simple linear regression allows us to estimate the values of *n* and log *K* as the slope and intersect of the linear dependence at each crystallization temperature. The characteristic time of crystallization τ_cr_ = 1/k can be determined for log[−ln(1 – α_DSC_(*t*))] = 0. The so-called Avrami plot of [P_666,2_][TFSI] determined from DSC measurements is shown in the inset of Figure 3c,d. Against theoretical prediction, the Avrami plot of [P_666,2_][TFSI] does not hold a linear nature across the time frame of the experiment.

**Figure 4:**
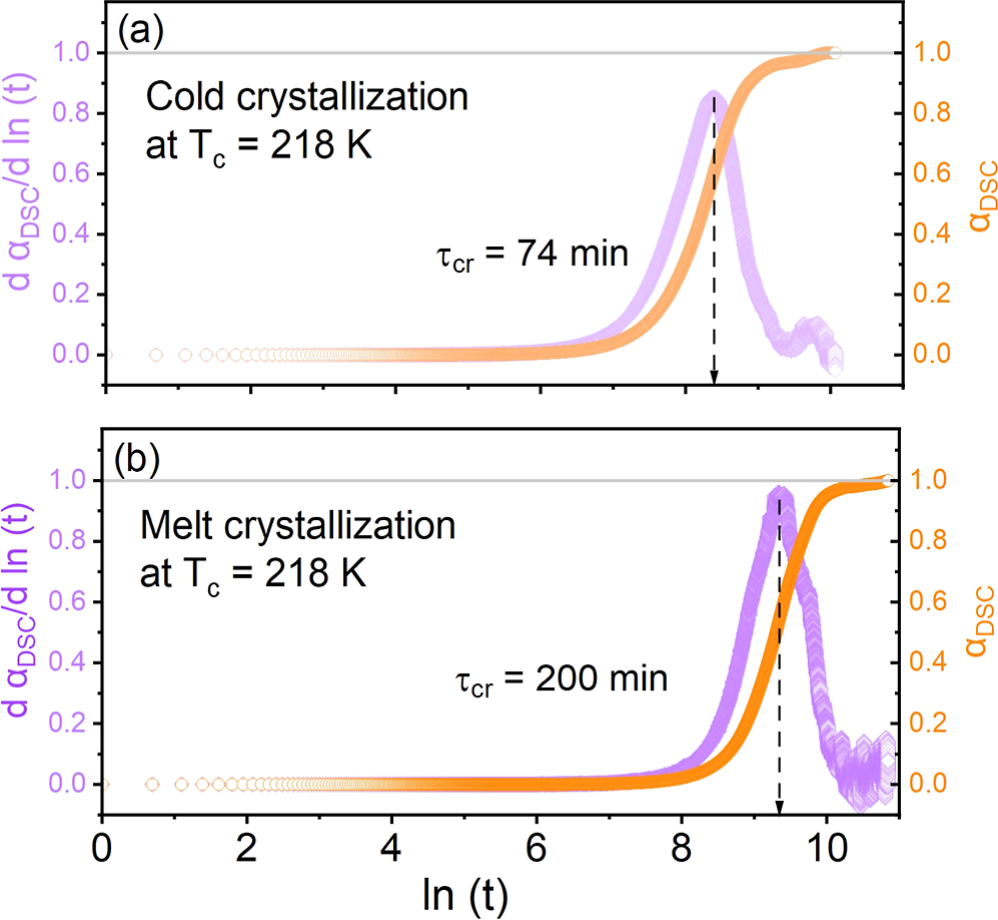
Avrami-Avramov plot of [P_666,2_][TFSI] obtained from the DSC measurements. Time evolution of the normalized degree of crystallization α_DSC_ and its first derivative with respect to the natural logarithm of the time at *T*_c_ = 218 K that was obtained from glass (a) and RT (b). Dashed arrows denote the characteristic crystallization time τ_cr_.

As the second model, a method proposed by Avramov et al.^41^ has been used (eq 4)
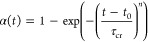
4in which the parameter *n* has the same meaning as in the Avrami model (eq 2). The key advantage of this approach is the possibility of avoiding measurement errors induced by the thermal instability of the sample at the beginning of the experiment and accurately estimating the crystallization induction time *t*_0_. Furthermore, the characteristic time for the isothermal crystallization, τ_cr_, can be easily determined from the maximum of the first derivate of normalized crystallization degree, dα(*t*)/d(ln(*t*)), plotted versus ln(*t*) (Figure 4a,b). The parameter *n* can be estimated as *n* = (α(*t*))’_max_/0.368. Substituting *t* – *t*_0_ by τ_cr_ in eq 4 gives α(τ_cr_) = 1–1/e ≈ 0.63. Consequently, to estimate *t*_0_, *t* can be found from the condition α(*t*)*’* = 0.63 and *t*_0_ is then calculated as *t*– τ_cr_.

A comparison of Avrami and Avramov characteristic parameters determined from DSC results recorded for [P_666,2_][TFSI] is given in Table 2.

**Table 2:** Comparison of Parameters Determined from Avrami and Avramov Models for Kinetics of Isothermal Crystallization of [P_666,2_][TFSI] (τ_cr_ Error within ±80 s, Log *k* Error within ±0.018, *n* Error within ±0.015, and *t*_0_ Error within ±50 s)

	DCS measurements									
[P_666,2_][TFSI]	*T*_c_ (K)	Avrami model				Avramov model				
		*n*	τ_cr_ (s)	k·10^–5^ (s^–1^)	log k	*n*	τ_cr_ (s)	*t*_0_ (s)	k·10^–5^ (s^–1^)	log k
cold crystallization	223	2.33	2316	43.2	–3.36	2.36	2368	55	42.2	–3.37
	218	2.30	4565	21.8	–3.66	2.32	4454	94	22.5	–3.65
	213	2.37	8428	11.8	–3.93	2.43	8992	577	11.1	–3.95
	208	2.61	17731	5.65	–4.25	2.62	17458	273	5.73	–4.24
	203	3.29	34967	2.86	–4.54	3.46	34076	768	2.93	–4.53
melt crystallization	220	1.91	53493	1.83	–4.74	2.46	50992	3000	1.96	–4.71
	218	2.52	12628	7.81	–4.11	2.54	12000	589	8.33	–4.08
	213	2.87	12689	7.93	–4.11	2.91	11585	1099	8.63	–4.06
	208	3.18	36109	2.78	–4.56	3.38	34513	1574	2.90	–4.54
	203									

**Figure 5:**
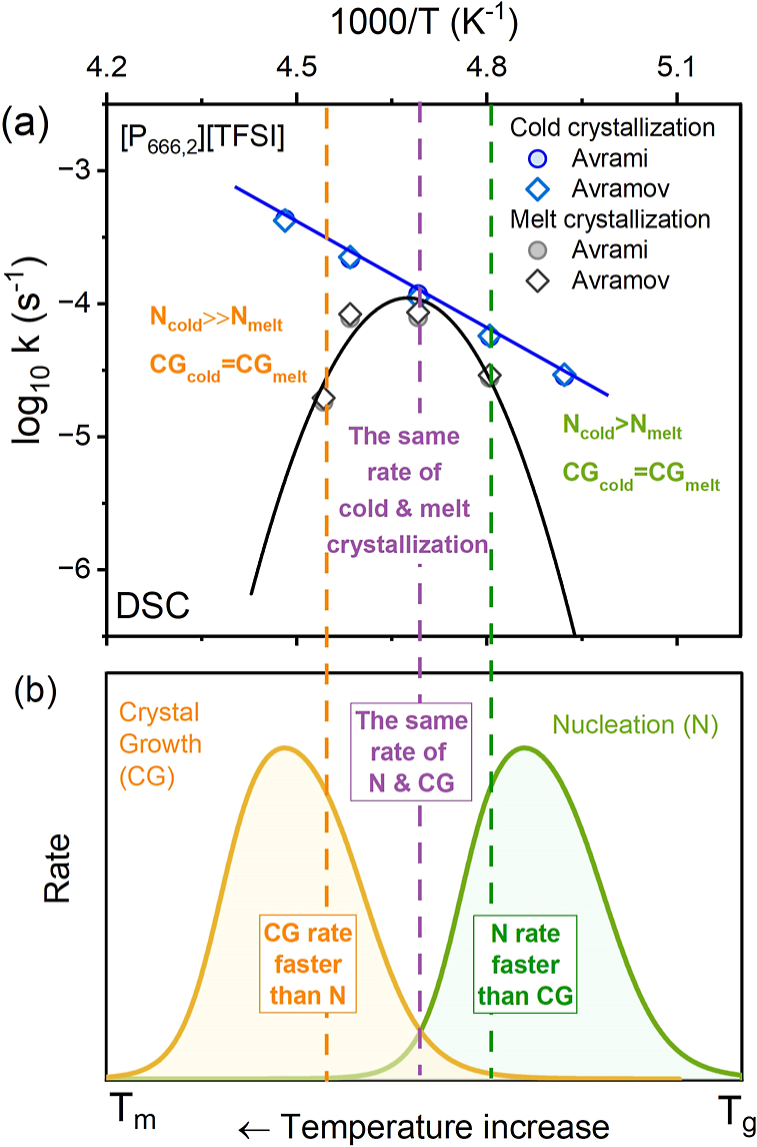
Panel (a) presents the temperature dependence of the crystallization rate obtained during cold and melt crystallization. Solid line represents the Arrhenius fit to the cold crystallization data. Panel (b) presents the schematic position of nucleation and crystal growth (CG) curves for [P_666,2_][TFSI].

For isothermal cold crystallization, the crystallization rate *k*, defined as *1/*τ_cr_, increased with increasing temperature. Consequently, from fitting the Arrhenius law (eq 5) to log *k* vs 1000/*T* dependence, the activation energy of cold crystallization for [P_666,2_][TFSI] could be determined as *E*_a_ = 51 kJ/mol (see Figure 5a)
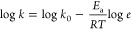
5where *R* is the gas constant and *k*_0_ and *E*_a_ are fitting parameters. In contrast, the rate of melt crystallization increased first and then decreased as the temperature increased, with the dependence log *k* vs 1000/*T* revealing a maximum of around 213 K.

These findings can now be related to the molecular structure of [P_666,2_][TFSI], with three relatively long hexyl chains on the cation and a flexible anion that can adopt a *cis* or *trans* conformation. It is generally known that the crystallization process involves two stages: (i) nucleation, N, and (ii) crystal growth, CG (Figure 5b),^12,42,43^ with each process reaching a maximum rate at a temperature somewhere between the glass-transition temperature and the melting point. While nucleation controls the crystallization at lower temperatures close to *T*_g_, CG dominates at higher temperatures close to *T*_m_ (Figure 5b). Avrami exponent *n* assumes the value of 4 for three-dimensional CG with a constant nucleation rate. A decrease to *n* = 3 informs of the existence of preformed nuclei or restricted dimensionality of crystallization (such as crystallization on the surface). Lower numbers may also be attributed to a decreasing nucleation rate, as crystallization releases heat, which causes a local temperature increase and suppresses crystallization.^14^

For cold crystallization, the parameters *n* (Avrami exponent connected to the time dependence of the nucleation rate and crystallization dimensionality), *k* (crystallization rate), log *k*, and τ_cr_ (characteristic time for the isothermal crystallization) were essentially independent of the chosen model. By supercooling to the glassy state and subsequent heating to *T*_c_^cold^ > *T*_g_, the IL has been passed through the nucleation maximum twice, creating a number of preformed nuclei for cold crystallization. For *T*_c_ = 213–223 K (higher temperature, close to *T*_m_), the Avrami exponent is *n* = ca. 2.35, suggesting preformed nuclei of crystallization and a decreasing nucleation rate. In this thermal range, CG is faster than N because there is enough kinetic energy for molecular diffusion to grow the lattice; combined with pre-existing nuclei, it results in high crystallization rates. Arguably, thermal energy from this fast crystallization could result in local heating of the IL, eliminating some nucleation sites and further contributing to low *n* values. For *T*_c_ < 213 K (closer to *T*_g_), the Avrami exponent increased with decreasing *T*_c_, reaching *n* = 2.6 at 208 K and *n* = ca. 3.35 at 203 K, corroborating with increasing nucleation rates in this region, dominating over molecular diffusion. It could be speculated that, at these lower temperatures, molecular diffusion is very slow, and the energy supplied by the ramped heating was used for subtle rearrangements of alkyl chains in cations and/or *cis*/*trans* switching in anions, leading to an increase in local ordering of [P_666,2_][TFSI] and thus - nucleation. This explains the longer time frame for such nucleation-driven crystallization to complete (Figure 3a).

For melt crystallization, there were significant discrepancies between the Avrami and Avramov models: the former predicted lower values of *n* at extreme temperatures (*n* = 1.91 at 220 K and *n* = 3.18 at 208 K), whereas the latter gave *n* = 2.46 at 220 K and *n* = 3.38 at 208 K (see Table 2 for a full comparison). Still, both models propose the same trend of increasing *n* with decreasing *T*_c_, analogous to cold crystallization. The core difference in melt crystallization is the absence of preformed nucleation sites. At *T* > 213 K, although the CG was fast in both melt and cold crystallization, the melt crystallization rate was lower due to the much smaller number of nuclei present. At *T*_c_ < 213 K, CG was very slow in both melt and cold crystallization, and crystallization was reliant on nucleation, N. Since some nuclei were precreated during cold crystallization (due to the crossing through the maximum of nucleation) in one sample but not in the other, log *k*^cold^ assumed higher values than log *k*^melt^.

Finally, the sample thermal history was not important for *T*_c_ at which N and CG rates were the same, i.e., 213 K for [P_666,2_][TFSI], as shown in Figure 5a. The cold and melt crystallization rates were equal, indicating the same contribution of nucleation and CG to the overall crystallization process.

To verify these results, gain better insight into the process dynamics, and determine how crystallization affects the conducting properties of [P_666,2_][TFSI], isothermal cold and melt crystallization experiments were subsequently monitored by BDS.

### Kinetics of Isothermal Crystallization of [P_666,2_][TFSI] by Dielectric Spectroscopy

BDS is a valuable tool for monitoring the crystallization process. Usually, the progress of crystallization is monitored by a decrease in relaxation strength that reflects a reduced number of mobile dipoles. However, in the case of ionic systems, the translational motions of charge carriers dominate both the imaginary and real parts of the dielectric function, ε″(*f*) and ε′(*f*). Therefore, the complex dielectric conductivity, σ* = ε_0_(Z*(*f*)·*C*_0_)^−1^, is commonly employed to express the properties of ILs.^44,45^ This representation was also selected to investigate the crystallization progress of [P_666,2_][TFSI]. The same experimental protocol was used for BDS measurements to compare the dielectric and calorimetric results quantitatively.

**Figure 6:**
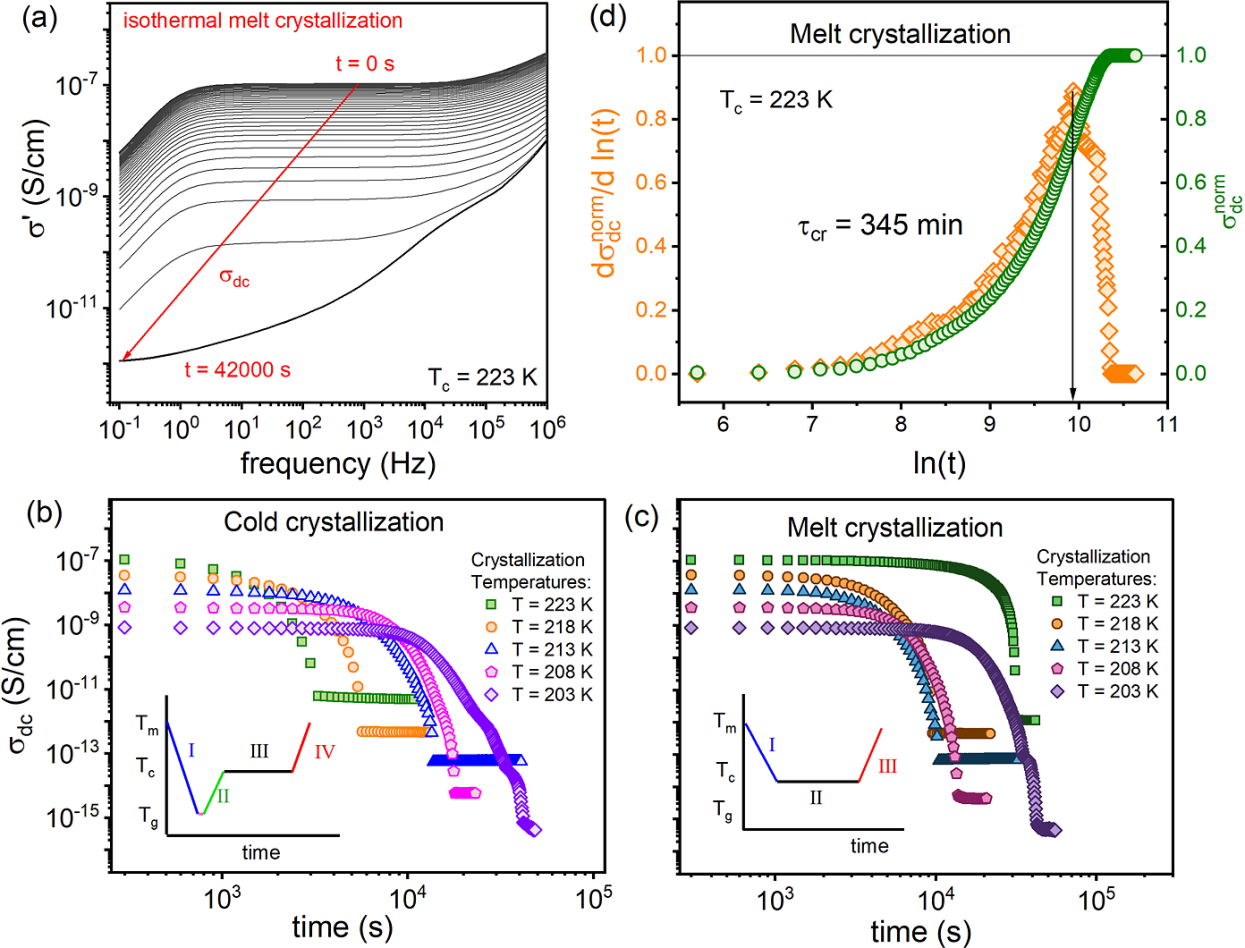
(a) Dielectric spectra of σ′(*f*) collected during isothermal melt crystallization of [P_666,2_][TFSI] at *T*_c_ = 223 K. Panel (b,c) presents the time evolution of σ_dc_ at various temperatures for [P_666,2_][TFSI] obtained during cold and melt crystallization, respectively. Insets show the experimental protocols. (d) Representative Avrami-Avramov plot of [P_666,2_][TFSI] obtained from the data presented in panel (a).

The representative frequency dependence of the real part of complex conductivity, σ′(*f*), measured during the melt crystallization at 223 K, is presented in Figure 6a. Each recorded curve, except the last one, showed three well-defined regions: (i) the low-frequency region; the area where the polarization effect takes place, (ii) the midfrequency plateau, also called dc-conductivity σ_*dc*_, and (iii) an approximate power-law increase at higher frequencies. A substantial drop in σ_dc_, observed in time-dependent measurement, is typical for crystallizing ionic systems because a rise in solid-state fraction reduces ions’ mobility, decreasing the number of species contributing to the σ_dc_. The time evolution of dc-conductivity recorded at various temperatures, with and without annealing in the glassy state, is presented in Figure 6b,c, respectively. In accordance with DSC studies, the cold crystallization time elongated as the temperature decreased for cold crystallization (Figure 6b), but not for melt crystallization (Figure 6c).

**Figure 7:**
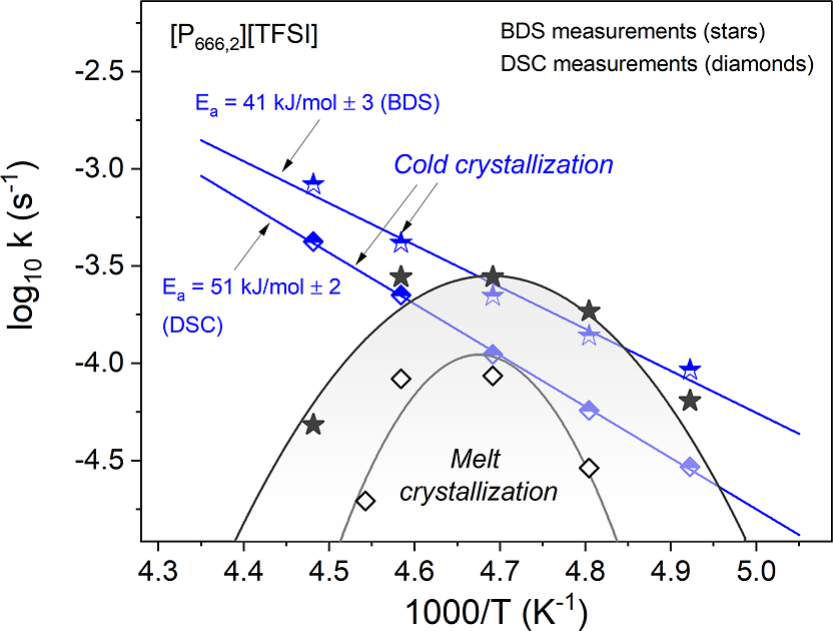
Comparison between the temperature dependence of log *k* obtained from the BDS and DSC methods for [P_666,2_][TFSI].

To characterize the crystallization kinetics of [P_666,2_][TFSI] in more detail, the time evolution of dc-conductivity (σ_dc_^norm^) behavior was normalized using the following formula
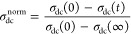
6where σ_dc_(0) and σ_dc_(∞) are the values of dc-conductivity at which crystallization begins and ends, respectively, whereas σ_dc_(*t*) denotes the value of σ_dc_ at a given crystallization time. The Avramov model was then selected to quantify the data. A representative Avrami-Avramov plot for melt crystallization kinetics at *T*_c_ = 223 K is shown in Figure 6d, while the parameters describing both cold and melt crystallization are summarized in Table 3. While it would be possible to compare and contrast numerical values of *n* and τ_cr_ derived from both methods, comparing these in graphic form has been found to be more effective. Thus, the temperature dependence of log *k*^BDS^ and log *k*^DSC^ obtained from the Avramov model has been plotted against 1000/*T* in Figure 7.

Aligned with the DSC results, log *k*^BDS^ depended linearly on *T*^–1^ for cold crystallization, and there was a maximum of log *k*^BDS^(*T*^*–1*^) at around 213 K found for melt crystallization. However, at each examined *T*, and irrespective of thermal history, the crystallization rate measured by BDS was faster than that determined from DSC measurements. Furthermore, the value of *E*_a_^BDS^ found for cold crystallization was 10 kJ/mol lower than that of *E*_a_^DSC^. The difference in *E*_a_ and crystallization rate between DSC and BDS has been frequently observed in the literature^46^ and can be attributed to different sample thicknesses, such as that in the former technique, which was 0.2 mm - much bigger than that in the DSC measurement. Furthermore, during the dielectric studies, the sample was placed between stainless-steel electrodes, inhibiting contact with air. Compared with DSC, the easier crystallization in the BDS system can be explained by the significant contribution of nucleation on the sample interface in BDS cells. However, the difference in *E*_a_ is within the experimental error and can be neglected.

A far more interesting aspect of the BDS study was the comparison between the homologous series [P_666,*n*_][TFSI] (*n* = 2, 6, and 8), as summarized in Table 3 and analyzed in-depth in the next section.

**Table 3:** Kinetic Parameters of Isothermal Crystallization [P_666,*n*_][TFSI] (*n* = 2, 6, and 8) Were Determined from Isothermal BDS Measurements Analyzed in Terms of the Avramov Model^a^

	*T*_c_ (K)	*n*	τ_cr_ (s)	*t*_0_ (s)	k·10^–4^ (s^–1^)	log *k*
[P_666,2_][TFSI]						
cold crystallization	223	1.46	1200	0	8.33	–3.08
	218	1.69	2400	0	4.17	–3.38
	213	1.99	4500	0	2.22	–3.65
	208	2.93	7200	0	1.39	–3.86
	203	3.22	10800	600	0.93	–4.03
melt crystallization	223	2.41	20700	2400	0.48	–4.32
	218	1.89	3600	0	2.78	–3.56
	213	2.12	3600	600	2.78	–3.56
	208	2.68	5400	600	1.85	–3.73
	203	3.12	15600	2100	0.64	–4.19
[P_666,6_][TFSI]						
cold crystallization	223	1.01	4800	300	2.08	–3.68
	218	1.59	7200	600	1.39	–3.86
	213	2.31	13200	1500	0.81	–4.12
	208	2.84	30000	2100	0.33	–4.48
[P_666,8_][TFSI]						
cold crystallization	223	2.60	76500	8100	0.13	–4.88
	218	2.26	37500	300	0.27	–4.57
	213	2.72	42000	600	0.24	–4.62
	208	2.19	68700	3900	0.15	–4.84

a (τ_cr_ error within ±80s, log *k* error within ±0.018, *n* error within ±0.015, and *t*_0_ error within ±50 s).

### Effect of Alkyl Chain Length on Isothermal Cold Crystallization and dc-Conductivity of Phosphonium ILs

**Figure 8:**
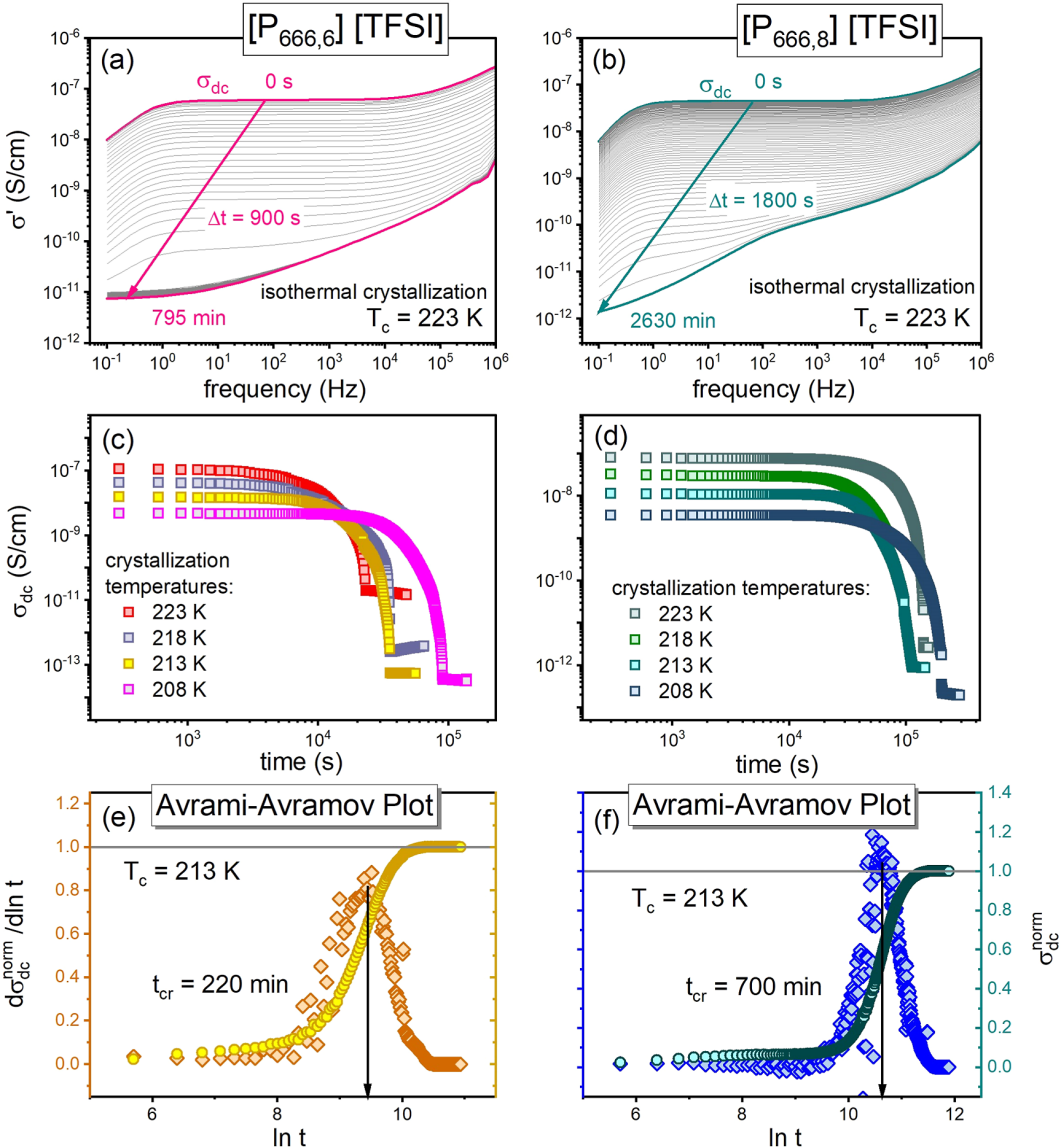
σ′(*f*) spectra collected during isothermal cold crystallization of [P_666,6_][TFSI] (a) and [P_666,8_][TFSI] (b) at *T*_c_ = 223 K. Panels (c,d) present the time evolution of σ_dc_ recorded at various temperatures of cold crystallization for [P_666,6_][TFSI] and [P_666,8_][TFSI], respectively. Panels (e,f) show representative Avrami-Avramov plots for these samples.

To examine the effect of alkyl chain length on the crystallization tendency of phosphonium ILs, isothermal crystallization measurements using the BDS setup were performed on the homologous series [P_666,*n*_][TFSI] (*n* = 6 and 8). Neither cold nor melt crystallization was observed for [P_666,12_][TFSI] during the heating/cooling scans or in isothermal studies. Since the annealing in the glassy state was found to speed up the crystallization process substantially, only the kinetics of cold crystallization were examined for [P_666,6_][TFSI] and [P_666,8_][TFSI]. After vitrification in a dielectric cell, the ILs were heated to *T*_c_ and maintained at this temperature until the end of the crystallization process. Representative spectra obtained during the time-dependent isothermal measurements at 223 K, for [P_666,6_][TFSI] and [P_666,8_][TFSI] are presented in Figure 8a,b. The Avramov model quantifies the results obtained under different temperature conditions (Figure 8c,d). The characteristic cold crystallization time τ_cr_ was determined from the Avrami-Avramov plot (see Figure 8e,f) at each examined *T*_c_ and collected with the other crystallization parameters in Table 3. Comparator plots for [P_666,2_][TFSI] are shown in Figure 6.

The cold crystallization time at 213 K increased from τ_cr_ = 220 min for [P_666,6_][TFSI] to τ_cr_ = 700 min for [P_666,8_][TFSI], which gives a 3-fold increase with an increase of alkyl chain length from 6 to 8 carbons. Completing this trend, the inclusion of the dodecyl carbon chain in [P_666,12_][TFSI] is so disruptive that no crystallization occurred. This is an interesting quantitative insight into ordering within long-chained phosphonium ILs. Literature reports on other properties being significantly affected by altering one chain on the phosphonium cation, for example, molar conductivity and ionicity in the [P_444,*n*_][TFSI] homologue series.^47^ Here, despite three hexyl chains, elongation of the fourth chain has a marked, disruptive effect on crystallization, significantly hindering the process’s kinetics, which has now been quantified for the first time.

**Figure 9:**
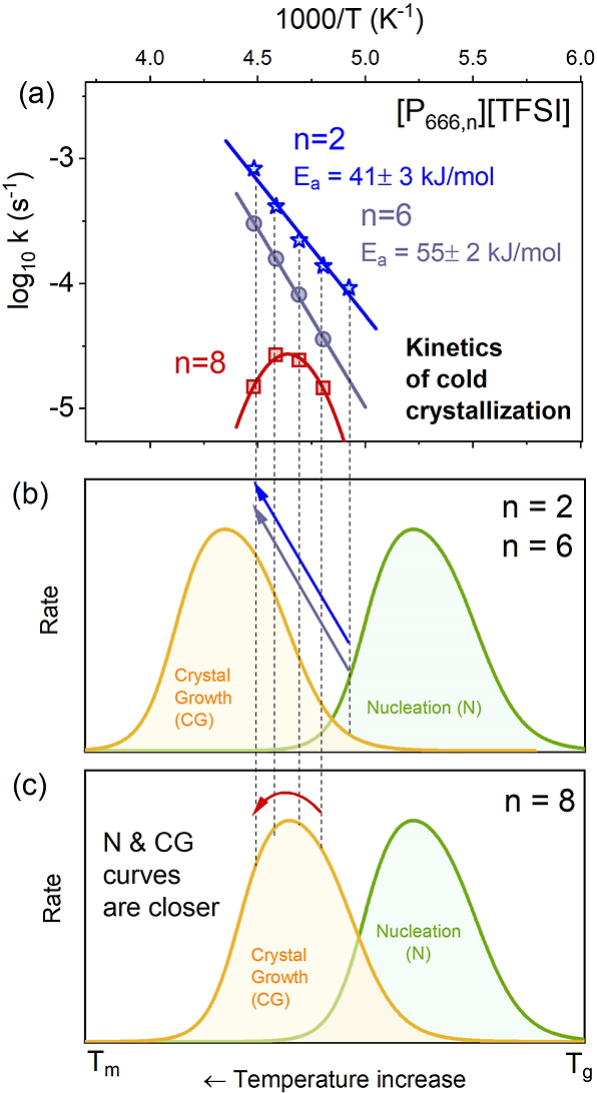
Comparison of log *k* (1000/*T*) for isothermal cold crystallization after annealing at the glassy state for [P_666,2_][TFSI], [P_666,6_][TFSI], and [P_666,8_][TFSI] measured from BDS.

To enable further comparison, the temperature dependence of log *k*^BDS^ for the homologous series, [P_666,*n*_][TFSI] (*n* = 2, 6, and 8) has been plotted in Figure 9. Irrespective of the *T*_c_ value, the log *k*^BDS^ values decreased substantially with an increase in the alkyl chain length. Furthermore, whereas the log *k* (*T*^–1^) plots for [P_666,2_][TFSI] and [P_666,6_][TFSI] were linear, albeit with higher activation energy for the latter (41 ± 3 vs 55 ± 2 kJ/mol, respectively), the Arrhenius law did not work for [P_666,8_][TFSI]. Specifically, the log *k* for this last IL featured a crystallization rate maximum at around 215 K (Figure 9a). Since crystallization of all the ILs has been initiated close to the glassy state, maximizing the nucleation rate, N, the difference in log *k* must result from the difference in CG time. Compared to [P_666,2_][TFSI] and [P_666,6_][TFSI], the nucleation and CG curves appear to be less separated in [P_666,8_][TFSI] (see Figure 9b,c).

Qualitative observations and numerical data recorded for the entire homologous series, [P_666,*n*_][TFSI] (*n* = 2, 6, 8, and 12) have led these authors to the speculative conclusion that crystallizations of phosphonium ILs differentiated by the length of one alkyl chain may differ in terms of rate but are the same in nature. That is, the entire series is thermodynamically capable of crystallization, and in each case, there is an expected maximum in log *k* vs 1000/*T*. However, the detection of the said maximum for *n* = 2 and 6 lies outside the examined temperature range.

**Figure 10:**
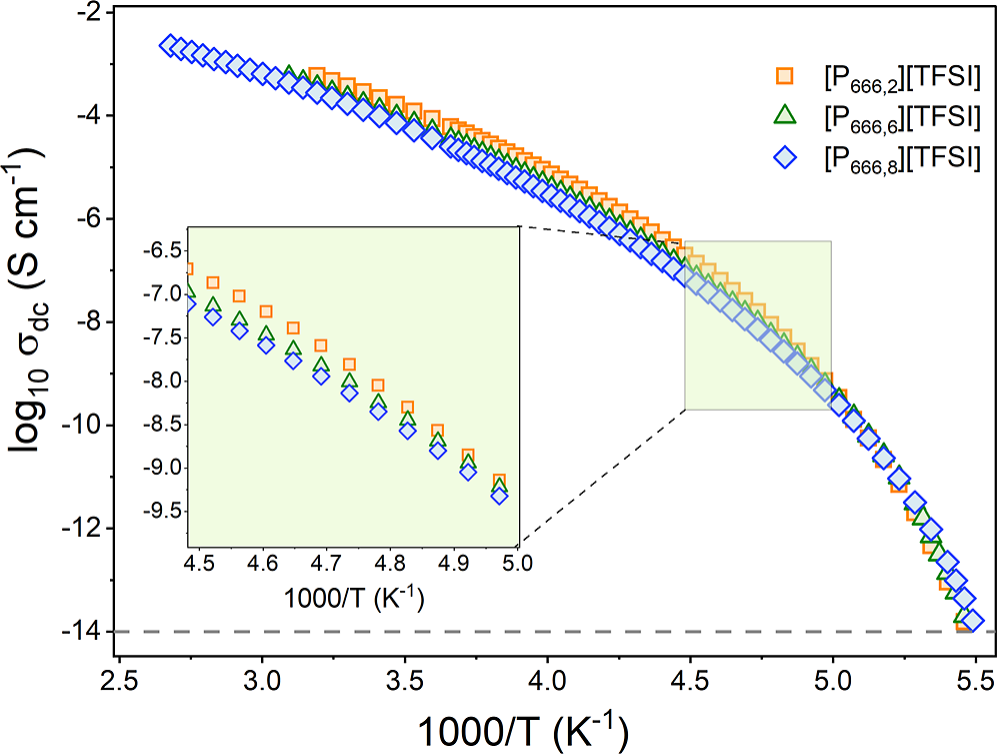
Temperature dependences of the dc-conductivity measured on cooling for [P_666,*n*_]^+^ ILs.

Initially, it was assumed that the difference in the kinetics of crystallization might be related to ionic mobility, as with an increased alkyl chain, the cations would be larger in volume and, therefore, less mobile. The temperature dependences of dc-conductivity have been examined to examine the mobility of the ions under given *T*_c_ conditions. As shown in Figure 10, in a supercooled liquid state, the σ_dc_ values at any given temperature were nearly identical for all ILs, in particular close to *T*_g_. This gives the fact that the difference in alkyl chain length for homologous ILs, [P_666,*n*_][TFSI] has no effect on ionic mobility. Thus, it has been established that the iso-conductivity conditions have been maintained during the isothermal crystallization studies. In consequence, in contrast to the original hypothesis, the crystallization of [P_666,*n*_]^+^ ILs is not governed by ion diffusion but by thermodynamic factors.

**Figure 11:**
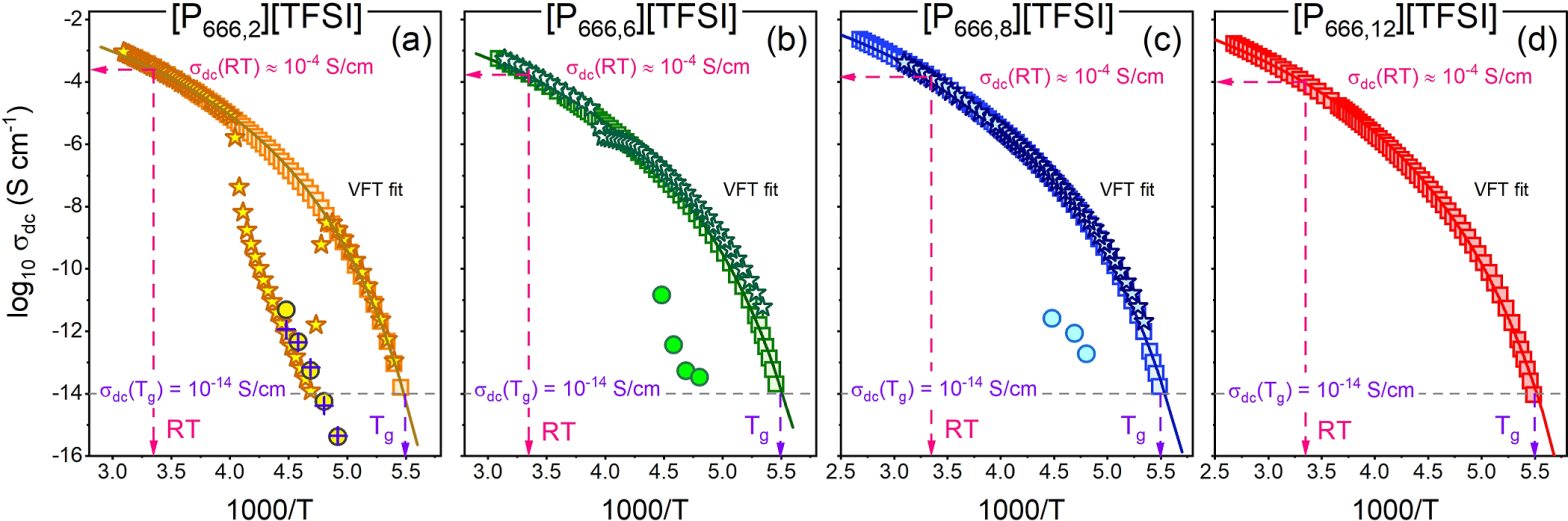
Temperature dependences of dc-conductivity measured for [P_666,n_]-based ILs using dielectric spectroscopy. Data obtained on the cooling run are denoted as squares. Stars present the dc-conductivity measured on heating. Circles denote the dc-conductivity of the crystalline phase obtained in the cold-crystallization process. Crosses denote the dc-conductivity of crystalline [P_666,2_][TFSI] obtained in the melt-crystallization process.

Finally, analysis of plots in Figures 6 and 8 revealed an interesting observation that at the end of the crystallization process, σ_dc_ was larger than 10^–14^–10^–15^ S/cm for all examined ILs, almost at every studied *T*_c_. To visualize this effect, we compared the σ_dc_ of crystalline materials to the σ_dc_ of supercooled ILs (Figure 11). For [P_666,*n*_][TFSI] (*n* = 2, 6, and 8), the temperature dependences of σ_dc_ recorded on cooling (squares) and heating (stars) scans have also been included. The σ_dc_ values of the crystalline phases obtained during heating and isothermal crystallization are pretty close and, except for a few points, larger than 10^–14^–10^–15^ S/cm. This indicates that some ion diffusion still exists in the crystalline state of the phosphonium ILs. In our earlier work,^31^ we identified a transient supercooled liquid state in some phosphonium ILs, comprising a “frozen” network of alkyl chains, forming corridors where the anions could move rapidly, resulting in high electrical conductivity. It seems plausible to suggest an analogous scenario in the crystalline phase of the [P_666,n_][TFSI] series, whereby immobile cations with interlocking alkyl chains form a matrix allowing for some anion mobility through the lattice, potentially enabled by the conformational flexibility of [TFSI]^−^. Indeed, in a study of a large family of phosphonium ILs by Hempelmann et al.,^47,48^ the [TFSI]^−^ anion has shown the highest self-diffusion coefficient despite being the largest (by volume) studied anion. Here, it has been demonstrated that the conductivity of the crystalline phase, like that of the supercooled liquid, had little dependence on the nature of the cation.

## Conclusions

Cold and melt crystallization of a series of phosphonium ILs, [P_666,*n*_][TFSI], have been investigated. Preliminary DSC studies showed all ILs to be relatively good glass formers, with similar *T*_g_ values and a decreasing cold crystallization tendency as the alkyl chain length increased.

Isothermal cold and melt crystallization of [P_666,2_][TFSI] using DSC has shown that the thermal history significantly impacts the crystallization kinetics; in samples that crystallized on heating (cold crystallization), the presence of nuclei has significantly sped up the process of crystallization. Irrespective of *T*_c_, cold crystallization was faster than melt crystallization at the same *T*_c_, with the exception of 213 K, at which a similar contribution of nucleation and CG to the overall crystallization resulted in equal cold and melt crystallization rates. This may be of great importance for fine control of crystallization phenomena, for example, in IL phase change materials.

Isothermal cold crystallization studies of [P_666,2_][TFSI], [P_666,6_][TFSI], and [P_666,8_][TFSI] by BDS, performed at the same temperatures and the same iso-conductivity conditions, revealed that the elongation of the alkyl chain in the cation increased the crystallization time. It has not been possible to crystallize [P_666,12_][TFSI] within the time frame of the experiment. It has been found that nucleation and CG curves are notably more separated for [P_666,2_][TFSI] and [P_666,6_][TFSI] when compared to [P_666,8_][TFSI], which resulted in the observable maximum in log *k* vs 1000/*T* plot of the latter. However, it has been concluded that a similar nature of non-Arrhenius plots could be, in theory, recorded for all ILs, including [P_666,2_][TFSI] and [P_666,12_][TFSI]. In conclusion, it has been speculated that while the kinetics of the process vary widely, the nature of crystallization in this homologue series remains the same.

Finally, non-negligible anionic conductivity in supercooled liquids and crystalline phases of [P_666,*n*_][TFSI] suggests a resemblance with our earlier work on the structure of transient, supercooled liquid phases in phosphonium ILs. Namely, it is suggested that phosphonium cations form immobile cationic networks of tangled alkyl chains, which remain porous enough–even in their crystalline state–that the flexible [TFSI]^−^ anions can permeate them.

## Supporting Information Available

DSC thermograms of examined systems and thermodynamic parameters obtained during the heating of crystalline [P_666,2_][TFSI] sample (PDF)
