# Supplementary material for: Crystallization Kinetics of Phosphonium Ionic Liquids: Effect of Cation Alkyl Chain Length and Thermal History
Source: J Phys Chem B. 2024 Jun 26;128(27):6610–21. doi: 10.1021/acs.jpcb.4c01720 (PMC11247483; doi:10.1021/acs.jpcb.4c01720)
Supplement: Supplementary file 1 — jp4c01720_si_001.pdf [file jp4c01720_si_001.pdf]

Supporting Information

Crystallization Kinetics of Phosphonium Ionic Liquids: Effect of Cation Alkyl Chain Length and Thermal History

B. Yao[1], V. Morales Alvarez[1], M. Paluch[1*], G. Fedor,[2] S. McLaughlin[2], A. McGrogan[2], M. Swadźba-Kwaśny[2], Z. Wojnarowska[1*]

[1]Institute of Physics, the University of Silesia in Katowice, 75 Pułku Piechoty 1A, 41–500 Chorzów, Poland

[2]The QUILL Research Centre, School of Chemistry and Chemical Engineering, The Queen's University of Belfast, David Keir Building, Stranmillis Rd, BT9 5AG Belfast, NI, UK.

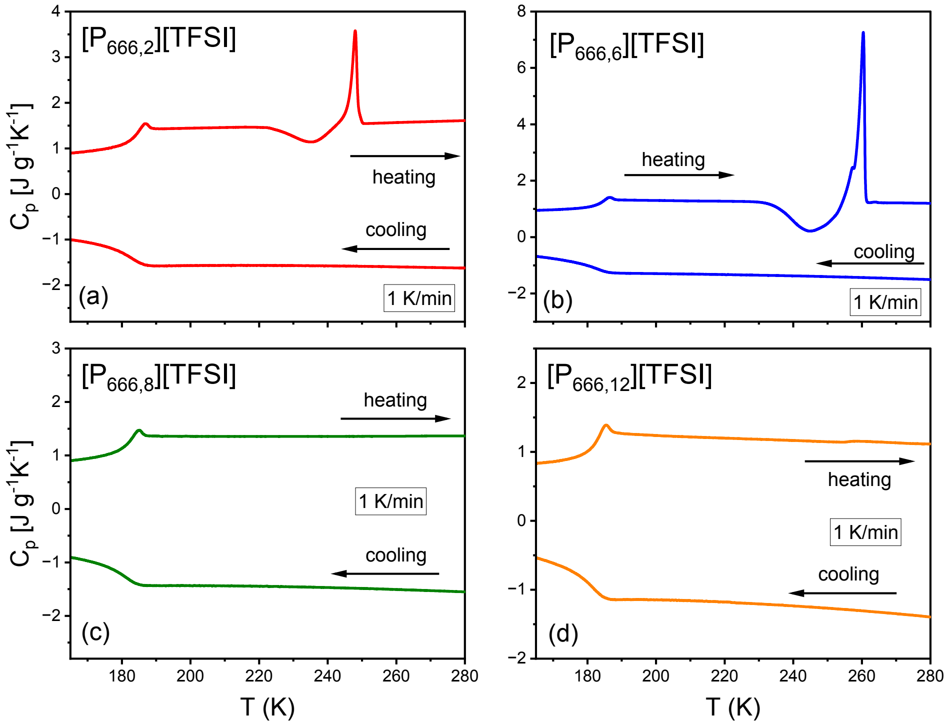

Figure S1 DSC thermograms collected during cooling and heating with a rate of 1 K/min for (a) [$P_{666,2}$][TFSI], (b) [$P_{666,6}$][TFSI], (c) [$P_{666,8}$][TFSI], (d) [$P_{666,12}$][TFSI].

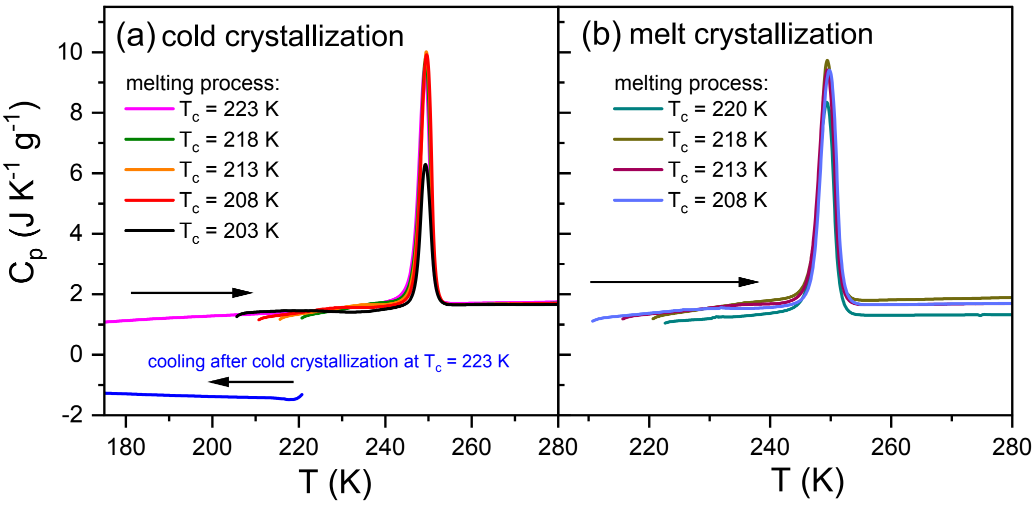

Figure S2 The DSC thermograms collected after cold crystallization **(a)** and melt crystallization **(b)** at different crystallization temperatures with a heating rate of 5 K/min.

Table S1 The enthalpy change ΔH and onset of melting temperature T_m during the heating process after crystallization for $[P_{666,2}]$[TFSI] measured from DSC

T_c (K)	Cold crystallization		Melt crystallization	
	ΔH (J g^{-1})	T_m (K)	ΔH (J g^{-1})	T_m (K)
220	-	-	23.75	246.4
223	25.42	246.2	-	-
218	25.35	246.5	25.32	246.6
213	25.45	246.6	25.44	246.3
208	25.44	246.7	25.52	246.7
203	14.57	246.7	-	-
